# *Spirulina platensis* Improves Mitochondrial Function Impaired by Elevated Oxidative Stress in Adipose-Derived Mesenchymal Stromal Cells (ASCs) and Intestinal Epithelial Cells (IECs), and Enhances Insulin Sensitivity in Equine Metabolic Syndrome (EMS) Horses

**DOI:** 10.3390/md15080237

**Published:** 2017-08-03

**Authors:** Daria Nawrocka, Katarzyna Kornicka, Agnieszka Śmieszek, Krzysztof Marycz

**Affiliations:** 1 Department of Experimental Biology, The Faculty of Biology and Animal Science, University of Environmental and Life Sciences, 27b Norwida Str., 50-375 Wroclaw, Poland; daria.k.nawrocka@gmail.com (D.N.); kornicka.katarzyna@gmail.com (K.K.); smieszek.agnieszka@gmail.com (A.Ś.); 2 Wroclaw Research Centre EIT+, Stablowicka Str. 147, 54-066 Wroclaw, Poland

**Keywords:** *Spirulina platensis*, equine metabolic syndrome, mesenchymal stromal cells, intestinal epithelial cells, oxidative stress, inflammation, insulin resistance

## Abstract

Equine Metabolic Syndrome (EMS) is a steadily growing life-threatening endocrine disorder linked to insulin resistance, oxidative stress, and systemic inflammation. Inflammatory microenvironment of adipose tissue constitutes the direct tissue milieu for various cell populations, including adipose-derived mesenchymal stromal cells (ASCs), widely considered as a potential therapeutic cell source in the course of the treatment of metabolic disorders. Moreover, elevated oxidative stress induces inflammation in intestinal epithelial cells (IECs)—the first-line cells exposed to dietary compounds. In the conducted research, we showed that in vitro application of *Spirulina platensis* contributes to the restoration of ASCs’ and IECs’ morphology and function through the reduction of cellular oxidative stress and inflammation. Enhanced viability, suppressed senescence, and improved proliferation of ASCs and IECs isolated from metabolic syndrome-affected individuals were evident following exposition to Spirulina. A protective effect of the investigated extract against mitochondrial dysfunction and degeneration was also observed. Moreover, our data demonstrate that Spirulina extract effectively suppressed LPS-induced inflammatory responses in macrophages. In vivo studies showed that horses fed with a diet based on *Spirulina platensis* supplementation lost weight and their insulin sensitivity improved. Thus, our results indicate the engagement of *Spirulina platensis* nourishing as an interesting alternative approach for supporting the conventional treatment of equine metabolic syndrome.

## 1. Introduction

According to the reports in the field of equine endocrinology, equine metabolic syndrome (EMS) has become a steadily growing life-threatening endocrine disorder associated with a constellation of disturbances comprising significant obesity, hyperinsulinemia, hyperlipidemia, and insulin resistance. It culminates with laminitis that is the second most common cause of death among horses [1]. One of the approaches in clinical and experimental settings for the treatment of this metabolic disease and its complications is cellular therapy involving the use of multipotent stem cells originating from a variety of adult tissues. Adipose-derived mesenchymal stromal cells (ASCs) are very common in both research and clinical practice. However, severe obesity together with regional adiposity, which are directly linked to elevated local and systemic inflammation, significantly attenuate the functionality of these cells and thus hinder therapeutic process in EMS horses [2]. Our previous data [3] pointed out the cytophysiological and molecular impairment of ASCs derived from EMS-diagnosed individuals (ASC_EMS_), questioned their clinical effectiveness. ASCs of EMS horses were characterized by elevated oxidative stress, senescent phenotype, enlarged nuclei and cell bodies, increased apoptosis, and reduced heterochromatin architecture. Moreover, upregulated oxidative stress was proved to induce inflammation in the intestinal epithelial cells (IECs) [4,5]. This is especially important in the context of a recently proposed concept, indicating intestinal inflammation as a mediator of obesity and insulin resistance development [6]. IECs are the first-line cells that are exposed to dietary compounds, especially in the small intestine, where they might promote early inflammatory changes, that work in favor of obesity and insulin resistance. It was shown that high fat diet contributes to elevated interferon-γ-induced genes expression, upregulation of TNF-α, as well as increased myeloperoxidase activity in IECs [7]. Therefore, IECs maintain essential immunoregulatory function that mediates the development and homeostasis of mucosal immune cells. Moreover, there is an increasing body of evidence indicating that the loss of intestinal homeostasis triggers systemic immune response, which in turn promotes the progression of metabolic disease [8]. However, it still needs to be elucidated how dietary compounds affect the expression of pro-inflammatory cytokines in IECs, which contribute to the development of chronic apoptosis.

One of the most important etiological factors in the course of EMS development is a high carbohydrate diet, which together with limited physical activity leads to obesity, increased systemic oxidative stress, and, finally, development of metabolic disturbances [9]. The oxidative stress that is linked to improper feeding strategy, particularly when there is excessive ingestion of carbohydrates without concomitant ingestion of antioxidant based ingredients, may contribute to systemic inflammation, enhanced senescence, and accelerated aging of adipose tissue [10]. Thus, searching for active ingredients that accelerate the healing process, seems to be fully reasonable. *Spirulina platensis* is the most commercialized blue-green algae species worldwide, willingly consumed due to widespread nutritional benefits. It is valued for high content of proteins, phytochemicals, as well as the variety of vitamins and minerals [11]. There is a constantly growing number of research studies providing evidence regarding its therapeutic benefits, including antioxidant, anti-inflammatory, immunomodulatory, anti-viral, anti-bacterial, neuroprotective, and hypolipidemic activities. Moreover, protective effects of *Spirulina platensis* against cancer, obesity, anemia, cardiovascular disease, and diabetes have been demonstrated [12,13]. Concomitantly, there are no reports on significant side effects associated with the use of microalgae as a dietary supplement. It seems most likely that the beneficial effects of Spirulina result from the content of phycocyanin and β-carotene, both with possible anticancer, anti-inflammatory, and free radical-scavenging properties. Moreover, the phenolic compounds found in Spirulina play a role in regulating redox signaling and therefore ameliorate the formation of reactive oxygen and nitrogen factors. Another substantial active compound of Spirulina is γ-linolenic acid (GLA) that has been reported as being essential for animals and humans. Anti-inflammatory, antioxidant, antibacterial, anti-fibrotic, anti-angiogenic, and cholesterol lowering properties of γ-linolenic acid have been demonstrated in multiple studies [11,14]. However, even though a number of studies addressing antioxidant, anti-inflammatory, and immunomodulatory properties of Spirulina have been done in recent years, the mechanism underlying the beneficial effects of Spirulina is not yet fully understood.

The aim of the current study was first of all to investigate whether in vitro application of *Spirulina platensis* could positively influence ASC_EMS_’ and IEC_EMS_’ oxidative stress and apoptosis levels, and thus improve their viability and function. Moreover, we employed the murine peritoneal macrophage model to demonstrate anti-inflammatory and immunomodulatory effects of *Spirulina platensis*. Then, the main goal of in vivo studies was to reduce the body weight and to investigate the influence of dietary protocol based on *Sprilulina platensis* supplementation on insulin resistance in EMS-affected horses.

## 2. Materials and Methods

Unless otherwise indicated, all chemicals and reagents used in this experiment were purchased from Sigma-Aldrich (Poznan, Poland).

All experimental procedures were approved by the II Local Ethics Committee of Environmental and Life Sciences University (Chelmonskiego 38C, 51-630 Wroclaw, Poland; decision No. 84/2012).

### 2.1. Quantification of Spirulina Platensis Functional Bioactive Components

#### 2.1.1. Determination of the Total Phenols Content (TPC)

Spirulina biomass was derived from Mühle Ebert Dielheim GmbH (MED, Dielheim, Germany). An amount of 0.5 g of the biomass was shaken for 1 h in the darkness with 10 mL of an 80% aqueous solution of methanol adjusted to a pH 1.5. The suspension was subsequently transferred to a homogenizer and homogenized for 1 min. The precipitate was centrifuged off (5 min, 6000 rpm) and the supernatant was subjected to further analysis. Total phenols content in the obtained extracts was determined spectrophotometrically. Then 1 mL of the sample was mixed with 1 mL of Folin–Ciocalteu reagent. After 3 min, 1 mL of saturated aqueous solution of Na_2_CO_3_ was added and the mixture was adjusted to a final volume of 10 mL with distilled water. The resulting solution was left for 90 min in the darkness to settle, and then filtered using syringe filters (PTFE membrane, pore size 0.45 μm). The absorbance was measured at 780 nm using quartz cuvette. The phenolic content was expressed as mg of gallic acid equivalent (GAE) per 100 mg of the sample.

#### 2.1.2. Extraction and Quantification of Fatty Acids

Total lipids were extracted from *Spirulina platensis* biomass according to a method of Folch et al. [15] with modifications. In brief, 1 g of algae dry mass was extracted with 10 mL of Folch mixture at room temperature for 24 h and sonicated for 1 h. The precipitate was filtered out using PTFE membrane (0.45 μm) to recover the liquid phase, and 1 mL of the solution was taken for further processing. The solvent was evaporated under vacuum at room temperature, and the residue dissolved in 0.5 mL of methyl tert-butyl ether (MTBE). 25 µL of internal standard solution (methyl undecanoate; 43 mg/10 mL MTBE) and 0.25 mL of trimethylsulfonium hydroxide (TMSH) 0.2 M in methanol was subsequently added. The analysis was performed using a Varian 450-gas chromatograph (GC) equipped with a flame ionization detector (FID). The fatty acids were separated in a Varian VF-WAXms column (30 m × 0.53 mm × 1 µm film thickness). Column temperature was programmed as follows: initial temperature was set at 50 °C (2 min), then raised from 50 °C to 250 °C at a rate of 10 °C/min, maintained at 250 °C for 23 min. Detector and injection chamber temperatures were set at 250 °C. Helium was used as a carrier gas at 1 mL/min flow.

#### 2.1.3. Analysis of Free and Protein-Bound Amino Acids

Free amino acids were extracted by mixing 1 g of dry alga with 10 mL of 1 M hydrochloric acid for 24 h at room temperature. Next, 0.25 mL of internal standard solution (norVal; 11 mg/10 mL) was added, the suspension was homogenized, and the precipitate separated by centrifugation. Then 0.1 mL of the extract was evaporated at 60 °C, and 0.05 mL of acetonitrile and 0.05 mL of functionalizing reagent *N*-tert-butyldimethylsilyl-*N*-methyltrifluoroacetamide with 1% tert-butyldimethylchlorosilane (MTBSTFA + 1% TBDMSCl) was added. The resulting mixture was heated at 100 °C for 1 h. Protein hydrolysis was carried out by heating 10 mg of investigated biomass with 1 mL of 6 M HCl at 110 °C. In order to enable analysis of acid-sensitive amino acids, hydrolysis in the presence of 1% phenol was additionally performed [16]. After completion of hydrolysis, 20 µL of the samples was collected, 10 µL of the standard (norVal, 11 mg/10 mL) was added, the mixture was evaporated (60 °C), and functionalization procedure was carried out as described above. Quantitative analysis was performed by GC/FID using Varian VF-5ms column (30 m × 0.53 mm × 0.5 µm film thickness). Helium was used as a carrier gas at a constant flow of 5 mL/min. The oven temperature program was set as follows: 5 min at 170 °C, heating at 4 °C/min up to 200 °C, maintenance at 200 °C for 3 min, increase to 300 °C at 4 °C/min, held at 300 °C for 20 min. Injection chamber and detector temperatures were 250 °C and 300 °C, respectively.

#### 2.1.4. Quantitative Analysis of Phycocyanin 

Prior to the analysis, 0.5 g of dry mass was extracted with the use of 1 M acetic acid (Ac), phosphate buffer (0.05 M, pH 6.8; PB) or 6 M hydrochloric acid (HCl). Then 10 mL of a solvent was used in each case. The samples were extracted for 24 h, sonicated for 1 h, and homogenized. The insoluble impurities were centrifuged off, and the resulting solution was filtered using a 0.45 µm membrane. The absorbance was measured at 615, 620, and 652 nm using cuvettes with optical path length of 1 cm. The phycocyanin content was calculated basing on the methods reported elsewhere [17,18].

#### 2.1.5. Determination of Vitamin C Concentration

Determination of vitamin C content was performed using the 2,6-dichlorophenolindophenol method (DIP) [19]. The standard curve was prepared for ascorbic acid in a concentration range 0 to 2 mg/100 mL. For the analysis, 0.5 g of the sample was extracted with 10 mL of extraction mixture (5 g of oxalic acid, 0.75 g of Na_2_EDTA in 1 dm^3^) using an ultrasonic extractor for 5 h at room temperature, and subsequently homogenized. Insolubles were centrifuged off and the solution was filtered with 0.45 µm membrane filters. DIP dye (0.02 mg/mL) was added directly to the extract in 5:1 (*v*/*v*) ratio. The absorbance was read spectrophotometrically at 520 nm using a quartz cuvette.

#### 2.1.6. Gas Chromatographic Assay of Tocopherol Content

Vitamin E (α-, β-, γ-, δ-tocopherol; Tc) content was determined by GC/FID chromatography. Quantification was based on the use of squalene as internal standard. For the analysis, 0.5 g of dry alga was shaken with 10 mL of 10% NaCl, 20 mL of hexane, 10 mL of methanol, and 10 µL of squalene solution (1.2 mg/mL) for 24 h at room temperature, and then sonicated for 1 h. The hexane phase was separated by centrifugation and evaporated to dryness. The remnants were dissolved in 25 µL of hexane, and 2 µL of a such prepared sample was fed to the injection chamber. Addition of antioxidant (butylated hydroxytolune; BTH) had no effect on the results. In turn, saponification of the samples led to a decrease in the tocopherols content. Varian VF-5ms column (30 m × 0.53 mm × 0.5 µm film thickness) was used for the chromatographic analysis, and the following conditions were applied: gas carrier: helium (flow 5 mL/min); injection chamber temperature: 250 °C; detector temperature: 340 °C; oven temperature program: 0.2 min at 110 °C, heating at 30 °C/min up to 140 °C, increase to 230 °C at 10 °C/min, held for 6 min, raise up to 300 °C at 10 °C/min and maintenance for 7 min.

### 2.2. In Vitro Study

#### 2.2.1. Animals Qualification

Before tissue collection, age-matched (9–14 years; mean ± S.D., 11.2 ± 1.7 years) donor horses of both sexes were classified into the EMS group (*n* = 6) or control group of healthy animals (*n* = 6). Detailed characteristic of the animals’ donating tissue for the purpose of the study is presented in Table 1. Assignation to the control or experimental group was performed on the basis of extensive interview with the animal keeper; measurement of bodyweight; estimation of body condition score and cresty neck scoring system; palpation and visual assessment of the hoof capsule; resting insulin and serum leptin levels; combined glucose-insulin test; and X-ray examination, as described by Basinska et al. [20].

#### 2.2.2. EqASC Isolation and Culture

The fragments of white subcutaneous adipose tissue were harvested form the horse tail base following standard surgical procedures and ethical norms as described previously [20]. Collected tissue specimens were placed in sterile Hank’s balanced salt solution (HBSS) supplemented with 1% antibiotic–antimycotic solution (Penicillin/Streptomycin/Amphotericin B, P/S/A). Adipose-derived mesenchymal stem cells’ isolation was performed following established protocol described in detail by Grzesiak et al. [21] and Marycz et al. [22]. Prior to cell isolation, tissue fragments were disinfected by brief immersion in a 70% ethanol solution and a subsequent thorough wash with HBSS containing 1% P/S/A, and disaggregated by mechanical mincing using sharp surgical scissors. Extracellular matrix was digested enzymatically with type I collagenase in a concentration of 1 mg/mL. Tissue homogenate was clarified by centrifugation at room temperature (1200× *g*, 10 min). Supernatant was discarded, and the resulting cell pellet further washed with HBSS, resuspended in culture medium and transferred to a T-25 polystyrene tissue culture flask. Prior to first passage, primary cell cultures were maintained in Dulbecco’s modified Eagle’s medium (DMEM) with nutrient F-12 Ham supplemented with 10% of Fetal Bovine Serum (FBS) and 1% P/S/A under optimal conditions (37 °C, humidified atmosphere of 5% CO_2_ in air). Upon reaching approximately 80% confluence, cells were gently detached using trypsin solution (TrypLE^TM^ Express, Life Technologies, Carlsbad, CA, USA) and transferred to T-75 flasks containing DMEM with 4500 mg/L glucose, 10% FBS, and 1% P/S/A to keep in culture. The media were refreshed every second day, and the cells were passaged when they reached 80%–90% confluence, 3 times prior to the experiment.

#### 2.2.3. IECs Isolation and Culture

Primary culture of equine small intestinal epithelial cells (IECs) was developed from abattoir-derived tissue of healthy and EMS-diagnosed horses. Collected small intestine specimens were transported in sterile HBSS containing 2% gentamicin solution. Prior to cell isolation, epithelium was separated from muscle tissue, and mechanically disintegrated. Resulting tissue biopsies were washed three times with HBSS and transferred into 2 mM EDTA solution in Ca^2+^ and Mg^2+^ free HBSS for digestion. The incubation was continued in a CO_2_ incubator at 37 °C for 30 min. Samples were monitored and intensively shaken using a vortex mixer periodically during the digestion process. Following completion of the incubation time, the samples were centrifuged at 300× *g* for 4 min at room temperature. Supernatants were discarded and cells pellets washed three times by consecutive suspension of the cells in HBSS and centrifugation. For the culture, cell pellets were transferred to T-25 flasks containing Ham’s F-12 medium supplemented with 10% of FBS, 1% P/S/A solution, and 25 μg amphotericin B per mL. The medium was changed partially (50%) every day during the first three days in culture, and totally every second day posteriorly. The cells were passaged when they reached 80% confluence, three times prior to the experiment.

#### 2.2.4. Immunophenotyping Characterization and Multipotency Assay

The immunophenotypic profile of isolated cells was analyzed by flow cytometry. Prior to the analysis, cells were detached from culture flasks using TrypLE^TM^ Express solution (Life Technologies), resuspended in HBSS containing 2% FBS, adjusted to a density of 1 × 10^6^, and aliquoted. CD45 cell-surface marker was identified in a one-step procedure with the use of mouse anti-equine CD45 APC-labelled monoclonal antibody (Novus Biologicals, Littleton, CO, USA). In turn, the expression of CD44, CD90, and CD105 markers was determined using the equine specific mouse primary monoclonal antibodies at 1/500 dilution (anti-CD44, R&D Systems, Minneapolis, MN, USA; anti-CD90, Abcam, Cambridge, UK; anti-CD105, Acris, Herford, Germany). All antibodies were added individually to the test tubes, and incubated for 30 min at 4 °C. Once the incubation was completed, the cells were washed three times by centrifugation in HBSS with 2% FBS, and subsequently stained with Alexa Fluor 488 fluorochrome-conjugated goat anti-mouse secondary antibody (Abcam). Acquisition of 10,000 single cell events was proceeded using Becton Dickinson FACS Calibur apparatus. Final data analysis was performed using Kaluza Analysis-Software (Beckman Coulter, Brea, CA, USA).

The multilineage differentiation potential of ASCs was evaluated as described previously [23]. A number of 1 × 10^4^ cells was seeded onto 24-well plates, and stimulated to differentiate into adipocytes, chondrocytes, and osteoblasts using commercially available kits (StemXVivo, R&D System). To induce adipogenesis, cultures were maintained under differentiation conditions for 9 days. The media were changed every 2 days. The effectiveness of the differentiation process was confirmed by visual analysis of intracellular lipid droplets formation performed using LipidTOX dye (Thermo Fisher Scientific, Waltham, MA, USA). In turn, osteogenic and chondrogenic stimulation was conducted for 14 days. Alizarin Red staining procedure was applied to evaluate mineralized nodule formation in the course of osteogenic stimulation, whereas 0.1% aqueous solution of Safranin O was used to visualize cartilage proteoglycans. Specimens were observed under an inverted epi-fluorescent microscope (Axio Observer A1, Zeiss, Oberkochen, Germany) and photographs were acquired with a Cannon PowerShot digital camera.

#### 2.2.5. Preparation of Spirulina Platensis Water Extract

*Spirulina platensis* used in this experiment was a food-grade powder purchased from Algamar S.A. (Pontevedra, Spain) and stored under dry and dark conditions upon arrival. To prepare the stock solution of Spirulina extract, 100 mg of *Spirulina platensis* powder was suspended in 10 mL of distilled water supplemented with 2% P/S/A and the extraction was carried out for about 10 min at room temperature. Once the extraction was complete, the resulting solution was further purified by filtration. To remove remaining algae material, the mixture was passed consecutively through 0.40 and 0.22 µm pore size syringe filters. The obtained clear Spirulina water extract was added directly to the culture media for cell stimulation.

#### 2.2.6. Cell Propagation with Spirulina Extract

Prior to the experiment, ASCs and IECs derived from healthy or EMS horses were seeded onto 24-well plate at the initial density of 2 × 10^4^ cells/well in 0.5 mL of culture media, and left overnight undisturbed to allow the cells to attach to the surface. Spirulina extract was added directly to the culture media in 1/4000 dilution. Stimulation lasted 24 h. Untreated cells served as controls.

#### 2.2.7. Visualization of Cell Morphology

ASCs’ and IECs’ morphology was examined by fluorescent microscopy. Actin cytoskeleton was detected and visualized using phalloidin staining for filamentous F-actin in fixed cells. To provide the correct fixation conditions for phalloidin binging, paraformaldehyde (PFA) was used as a fixative. The preparation of cells was performed following the applicable instructions, and included the following steps: (i) triple wash with HBSS, (ii) fixation with ice-cold 4% PFA diluted in PBS—overnight incubation at 4 °C followed by extensive wash with HBSS, (iii) permeabilization with 0.1% Triton X-100 in HBSS for 15 min at room temperature, (iv) a final wash with HBSS, (v) staining with atto-488-labeled phalloidin at 1:800 dilution in HBSS for 40 min at room temperature in the dark. Nuclei were counterstained with diamidino-2-phenylindole (DAPI) solution 1:1000 in HBSS by 5 min incubation in the dark at room temperature. The cells were observed using epi-fluorescent microscopy.

#### 2.2.8. Cell Viability Assay—TOX8

The viability of ASCs was assessed with resazurin-based fluorescent dye (TOX8 In Vitro Toxicology Assay Kit). The procedure was performed according to the protocol provided by the supplier. For the assay, media from over cell cultures were removed and replaced with 350 μL of 10% *v*/*v* dye solution in DMEM/F-12 + 10% FBS. Incubation was carried out in the CO_2_ incubator at 37 °C for 2 h. Supernatants were subsequently collected, transferred to a microtiter plate in a volume of 100 μL, and subjected to spectrophotometric measurement using a 96-well microplate reader (Spectrostar Nano, BMG Labtech, Ortenberg, Germany). Additional wells containing complete medium without cells served as blank. Reduction of the dye was evaluated at 600 nm wavelength and 690 nm reference wavelength.

#### 2.2.9. BrdU Cell Proliferation Assay

Proliferative activity of IECs was estimated by quantification of BrdU incorporation into the newly synthesized DNA of proliferating cells. The assay was performed using the BrdU Cell Proliferation ELISA Kit (Abcam) following the manufacturer’s protocol. Briefly, prior to the experiment, cells were seeded onto a 96-well plate at a concentration of 1 × 10^4^ cells in 100 μL/well of culture media. Three of the wells on the plate were assigned to BrdU-treated group, another 3 served as an assay background that contain cells but did not receive the BrdU reagent, the other wells were set as controls that did not receive cells, but media alone. For the assay, diluted BrdU reagent was added directly to the culture medium in a volume of 20 μL. Incubation was carried out for 24 h at 37 °C in CO_2_ incubator. The cells were subsequently fixed, permeabilized, and the DNA was denaturated by incubation with the provided fixing solution. Incorporated BrdU was detected with anti-BrdU monoclonal detector antibody. Horseradish peroxidase-conjugated goat anti-mouse IgG was used as a secondary antibody. Color reaction was developed with peroxidase chromogenic substrate tetra-methylbenzidine (TMB). The coloured reaction product was measured using a spectrophotometric mictrotiter plate reader (BMG Labtech) set at a single wavelength of 450 nm.

#### 2.2.10. Evaluation of Cellular Senescence and Apoptosis

Quantitative evaluation of cellular senescence in ASCs and IECs was carried out by senescence-associated β-galactosidase assay using a Senescence Cells Histochemical Staining Kit. Briefly, cells were rinsed twice with HBSS and fixed with the provided formaldehyde-based fixation buffer for 6 min at room temperature, then thoroughly washed with HBSS, and stained with a solution containing 5-bromo-4-chloro-3-indolyl-b-d-galactopyranoside (X-gal). Following overnight incubation, supernatants were collected and measured for absorbance at 420 nm, whereas senescent cells were identified as blue-stained cells under the inverted fluorescent microscope.

Additionally, the viability of cells in the examined groups was assessed with Cell Stain Double Staining Kit. Calcein A.M. reagent and nuclei staining dye Propidium Iodide (PI) were used for simultaneous fluorescent staining of viable and dead cells. The cells were imaged using an epi-fluorescent microscope, and the viability was assessed based on the staining results and calculation of percentage of dead cells among the entire population. All procedures were performed in accordance with the protocols provided by the manufacturers.

#### 2.2.11. Flow Cytometric Analysis of JC-1 and H2DCF-DA Stained Cells

Mitochondrial depolarization that occurs in the early stages of apoptosis was detected in ASCs and IECs at a single-cell level by the means of flow cytometric mitochondrial membrane potential determination by JC-1 fluorescent probe (Life Technologies). In turn, intracellular reactive oxygen species (ROS) were estimated by using H2DCF-DA reagent (Life Technologies).

For the analysis, cells of each investigated group were detached from the culture flasks, resuspended in HBSS, and evenly divided into six aliquots (six test tubes). Then 5 μM JC-1 or 10 μM H2DCF-DA were added directly to the cell pellet (*n* = 3 per stain). Incubation was carried out for 30 min at 37 °C. When the incubation was over, cells were washed twice in HBSS supplemented with 2% FBS and subjected for FACS analysis; 1 × 10^4^ events were collected for each analysis. Results were expressed as the percentage of deteriorated mitochondria that exhibit green fluorescence coming from monomers of JC-1 at depolarized membrane potentials, or as the percentage of ROS-positive cells.

#### 2.2.12. Evaluation of Extracellular Oxidative Stress

For the quantification of extracellular oxidative stress level, ASCs and IECs were cultured in designated growth media without phenol red in the presence or absence of Spirulina. Nitric oxide concentration in cell-free supernatants was measured using commercially available Griess reagent kit (Life Technologies), whereas superoxide dismutase activity was detected with SOD Assay Kit. Procedures were carried out in triplicates, following manufacturer’s recommendations.

#### 2.2.13. Collection and Stimulation of Murine Peritoneal Macrophages

Resident peritoneal cells’ isolation was performed according to the protocol established and described by Ray and Dittel [24]. In brief, untreated 8 weeks old Swiss mice were euthanized by CO_2_ asphyxiation, sprayed extensively with 70% EtOH and mounted onto styrofoam block. The outer skin of the peritoneum was cut and pulled back to expose the lining inner skin. The peritoneal cavity was then carefully flushed with 6 mL of ice-cold FBS (3%) enriched HBSS. Collected cell suspension was deposited into 15 mL falcon collection tubes and kept on ice. In the case of blood contamination, peritoneal wash fluid was discarded. Following isolation, peritoneal exudate cells were centrifuged (10 min, 400× *g*, 4 °C), re-suspended in cold DMEM with 4500 mg/L glucose, 10% FBS, 1% P/S/A, and 2 mM l-glutamine, counted, adjusted to a density of 1 × 10^6^ cells/mL, and seeded onto a 24 well-plate in a volume of 500 µL/well. The cells were allowed to adhere to the surface of the culture vessels by culturing them for 18 h at 37 °C in 5% CO_2_ humidified atmosphere. Non-adherent cells were removed by a gentle wash with warm HBSS afterwards. Greater than 95% of adherent cells were expected to be macrophages at the time. Culture media of designated groups were supplemented with Spirulina extracts at 1/4000 dilution and the culture was carried out for next 24 h. LPS was added to the appropriate culture media afterwards at a concentration of 1 µg/mL and the experiment was continued for another 24 h. The culture media were subsequently collected for further analysis of the macrophages’ secretory activity, and the cells were lysed directly in the culture dish by adding TRI Reagent^®^ (300 µL/well), and evaluated for inducible nitric oxide synthase (iNOS), as well as TNF-α and IL-6 pro-inflammatory cytokines gene expression.

#### 2.2.14. Determination of Nitric Oxide Production by Peritoneal Macrophages

The accumulation of NO in cell-free supernatants from over peritoneal macrophages cultured in the presence or absence of Spirulina extract and/or lipopolysaccharide was quantitatively measured using commercially available assay system—Griess reagent kit (Life Technologies). The procedure was performed following the protocol provided with the kit. The absorbance was read at 548 nm wavelength using a 96-well microplate reader (Spectrostar Nano, BMG Labtech, Ortenberg, Germany).

#### 2.2.15. Measurement of TNF-α and p53 Levels by ELISA

The concentration of TNF-α and p53 protein in cell-free culture supernatants was determined by solid phase ELISA using the Mouse TNF-α Quantikine ELISA Kit (R&D Systems) and Horse p53 ELISA Kit (MyBioSource, San Diego, CA, USA), respectively. All steps in the procedure and calculations were carried out according to the manufacturer’s instruction. The absorbance was measured with a 96-well microplate reader (Spectrostar Nano, BMG Labtech) at 450 nm.

#### 2.2.16. Analysis of Gene Expression

Expression of apoptosis-related p21, p53 and Bax genes, PINK1/Parkin mitochondrial quality control genes, as well as inflammatory mediators (iNOS) and cytokines (TNF-α, IL-6) was investigated by quantitative reverse-transcription real time polymerase chain reaction (qRT-PCR). Upon the completion of the experiments, the cells were homogenized in TRI Reagent^®^. Total RNA was isolated by phenol-chloroform extraction and ethanol precipitation, as described by Chomczynski and Sacchi [25]. Extracted total RNA was further aliquoted in DEPC-treated water and submitted for quantitative and qualitative analysis using a nanospectrophotometer (WPA Biowave II) at 260/280 nm wavelengths. Prior to gene expression profiling, reverse transcription reaction together with genomic DNA digestion was carried out using PrimeScript™ RT reagent Kit with gDNA Eraser (Perfect Real Time; Takara Clontech, Otsu, Shiga, Japan). Then 150 ng of totalRNA was used as a template for a single reaction. The procedure was performed according to the protocol provided by the manufacturer, and involved the use of T100 Thermo Cycler (Bio-Rad, Hercules, CA, USA). All qRT-PCR reactions were carried out on 2 µL of cDNA in a 20 µL reaction volume using 500 nM of each specific primer. Primer sequences used for amplification of selected target genes are listed in Table 2. The reactions were performed using SensiFast SYBR & Fluorescein Kit (Bioline, Cincinnati, OH, USA) and a CFX ConnectTM Real-Time PCR Detection System (Bio-Rad) at the following thermal cycling conditions: initial enzyme activation at 95 °C for 5 min, 45 PCR cycles of 95 °C for 15 s for denaturation, optimal primer annealing temperature for 30 s, and 72 °C for 15 s for elongation. Melting-curve analysis was subsequently performed to verify the specificity of the product. The mRNA levels were normalized relative to the GAPDH or β-Actin housekeeping genes. The data were analyzed by the 2^−ΔΔ*C*T^ method.

#### 2.2.17. Statistical Analysis

Statistical analyses were performed on the basis of three independent experiments. The results shown in figures represent mean values ± standard deviation (SD). The data were analyzed by one-way analysis of variance (ANOVA) followed by Tukey’s post hoc multiple comparison test using GraphPad Prism 5.01 (San Diego, CA, USA). *p* values less than 0.05 were considered statistically significant. Hash (^#^) and Asterisk (*) sign indicated statistical significance in ASCs and IECs healthy control versus EMS control or EMS control versus EMS sprirulina-treated group, respectively. *p* values less than 0.05 (*p* < 0.05) were summarized with one asterisk/hash (*/^#^) *p* < 0.01 with two asterisks/hashes (**/^##^) *p* < 0.001 with three asterisks/hashes (***/^###^).

### 2.3. In Vivo Study 

#### 2.3.1. Animals Classification

Horses (*n* = 18) of different breed (Silesian Breed, Haflingers), both sexes, age range 8–14 years were involved in the study. The experimental horses were chosen on the basis of clinical examination and detailed interview with the owners, as described in Section 2.2.1. Animals qualified for the study were divided into three groups: (i) control group of healthy horses (*n* = 6)—received control feed A; (ii) control group of EMS-diagnosed horses (*n* = 6) also fed with control feed A; (iii) experimental group of EMS-diagnosed horses (*n* = 6) that were fed with pelleted Spirulina—test feed B. During the experiment animals spent the majority of the day outside on a sandy paddock, but were excluded from pasture. One hour per day of moderate intensity exercise (longing) was provided.

#### 2.3.2. Dietary Protocol

The tested Spirulina (feed B, pelleted Spirulina), as well as control feed (feed A, pelleted hay) were manufactured by Mühle Ebert Dielheim GmbH (MED, Dielheim, Germany). Horses classified in the experimental group received 500 g of feed B once a day, whereas healthy or EMS-diagnosed horses assigned to the control reference groups received the same amount of control feed A. The daily dietary protocol included timothy grass hay (1.5% per 1 kg of body weight) and water ad libitum in the case of all groups involved in the study. Animals did not receive any commercial feed or mineral additives during the experiment. Clinical examination together with body weight measurement of each individual was performed before and at the end of the 3-month dietary trial.

#### 2.3.3. Statistical Analysis

Statistical significance was determined by one-way ANOVA with Tukey’s post hoc test using GraphPad Prism 5.01 (San Diego, CA, USA). *p* values less than 0.05 were considered statistically significant. Asterisk (*) sign indicated statistical significance between control and experimental groups of EMS-diagnosed horses. *p* values less than 0.05 (*p* < 0.05), *p* < 0.01 and *p* < 0.001 were summarized with one (*), two (**) or three asterisks (***), respectively.

## 3. Results

### 3.1. Spirulina Platensis as a Source of Bioactive Compounds

A number of quantitative analyses was conducted to provide information regarding the composition of *Spirulina platensis* in terms of its bioactive compounds. To determine the fatty acids profile of investigated samples, Spirulina extract was checked for 17 different fatty acids. The data confirmed the presence of 13 of them (Table 3). Palmitic, linoleic, and γ-linolenic acids were identified as the major ones. They amounted to ca. 2423, 1053, and 744 mg/100 g of dry Spirulina, respectively. In addition, the presence of palmitoleic acid, stearidonic acid, coenzyme A, stearic, myristic, lauric, arachidic, eicosadienoic, caprylic, and caprid acids was confirmed. No trace of myristoleic, pentadecylic, α-linolenic, and behenic acids was found.

Moreover, CG analysis was performed to quantify 20 free and bound amino acids, including alanine, glycine, valine, leucine, isoleucine, asparagine, aspartic acid, glutamine, glutamic acid, lysine, arginine, histidine, phenylalanine, tyrosine, tryptophan, serine, threonine, methionine, cysteine, and proline. The results are presented in Table 4.

Separation profile revealed the presence of 15 free-form amino acids in quantities from 20 to 437 mg/100 g of algae dry mass. Unbounded asparagine, arginine, tryptophan, serine, and cysteine were not detected, indicating that these amino acids are solely incorporated into proteins or peptides.

Phycocyanin with its anti-oxidant and anti-inflammatory properties constitutes the major active compound of *Spirulina platensis.* We determined the concentration of phycocyanin in Spirulina samples extracted with three different solvents: acetic acid, phosphate buffer, and hydrochloric acid. The content of the phycocyanin in the sample extracted with phosphate buffer was 266 ± 23 mg/100 g. It was not detected in the two other extracts. Moreover, Spirulina samples were tested for total phenols. Total phenolic compounds were measured with the use of Folin–Ciocalteu reagent. The mean concentration was expressed as equivalents of gallic acid and amounted to 176 ± 5.0 mg/100 g of the sample. Other important active constituents of *Spirulina platensis* are vitamins, including vitamin C and tocopherols—naturally occurring chemical compounds with vitamin E activity. Two forms of tocopherols were found in investigated samples—α-tocopherol (α-Tc) that amounted to 2.43 ± 0.21 mg/100 g of the sample, and γ-tocopherol (γ-Tc) at a concentration of 1.07 ± 0.21 mg/100 g. The mean ascorbic acid content in *Spriulina platensis* was 18.37 ± 0.97 mg/100 g of dry algae.

### 3.2. Immunophenotype and Multipotent Properties of Isolated Cells

**Figure 1:**
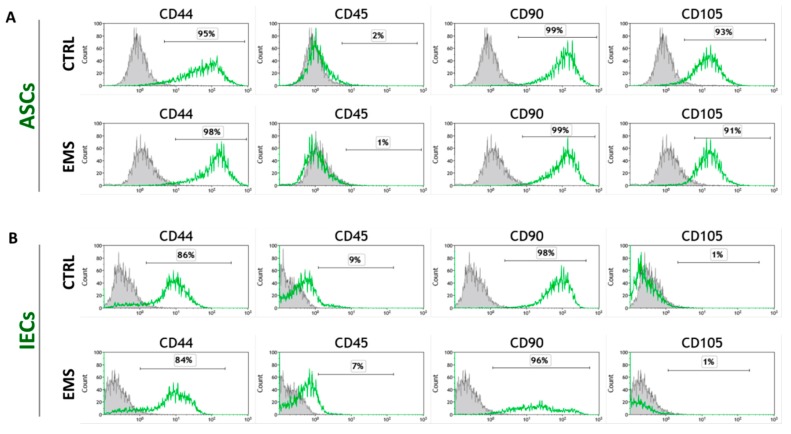

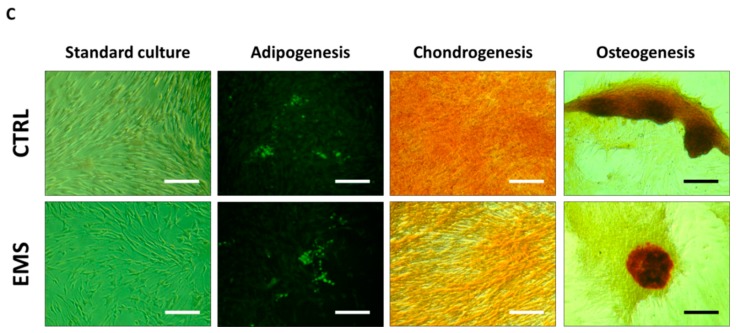
Analysis of cell-surface markers expression and multipotency assay. Representative flow cytometry histograms of passage 3 equine adipose-derived mesenchymal stromal cells (ASCs) (**A**) and intestinal epithelial cells (IECs) (**B**) originating from healthy (CTRL) and EMS-diagnosed (EMS) horses by fluorescence-activated cell sorting (FACS). Unstained cells provided negative control for the analysis (grey peaks). ASCs derived from both healthy and EMS donors were characterized by the expression of CD44, CD90, and CD105, and lack of CD45 hematopoietic marker. IECs displayed no expression of CD105 marker. Representative images from trilineage differentiation induced at passage 3 ASCs (**C**). Multilineage differentiation capacities were assessed in ASCs cultured in appropriate culture media. Lipid droplets accumulation in response to adipogenic stimulation was confirmed with LipidTOX dye; cartilage formation in chondrogenic cultures was evaluated using Safranin O reagent, while mineral depositions obtained under osteogenic conditions were detected with Alizarin Red. Cells cultured under standard conditions provided control for evaluation of differentiation effectiveness. Magnification 100×, scale bars: 250 μm.

For phenotypic characterization of isolated cell populations, the surface marker expression was analyzed by flow cytometry of ASCs and IECs derived from healthy and EMS-diagnosed horses. Both ASC cell populations (ASC_CTRL_ and ASC_EMS_) fulfilled the requirements of the International Society for Cellular Therapy in assuring MSC identity [26] by displaying plastic adherent growth and exhibiting high CD44, CD90, CD105 expression, and lack of CD45 hematopoietic marker (Figure 1A). CD105 is an endoglin that is abundantly expressed in tissues undergoing angiogenesis. Interestingly, ASCs have been shown to express low levels of CD105 just after isolation and become increasingly CD105 positive over time in culture [27]. In contrast, intestine epithelial cell (IEC) phenotype was characterized by lack of CD105 marker expression (Figure 1B). Additionally, multipotent nature of ASCs was confirmed by positive results of differentiation into osteoblast, chondrocytes or adipocytes in vitro, as demonstrated by specific lineage staining (Figure 1C).

### 3.3. Cell Exposition to Spirulina Extract Improves ASCs’ and IECs’ Morphology and Proliferative Activity

Morphological characteristics and viability of investigated cell populations were assessed after 48 h of culture, including 24 h of cell stimulation with Spirulina extract.

**Figure 2:**
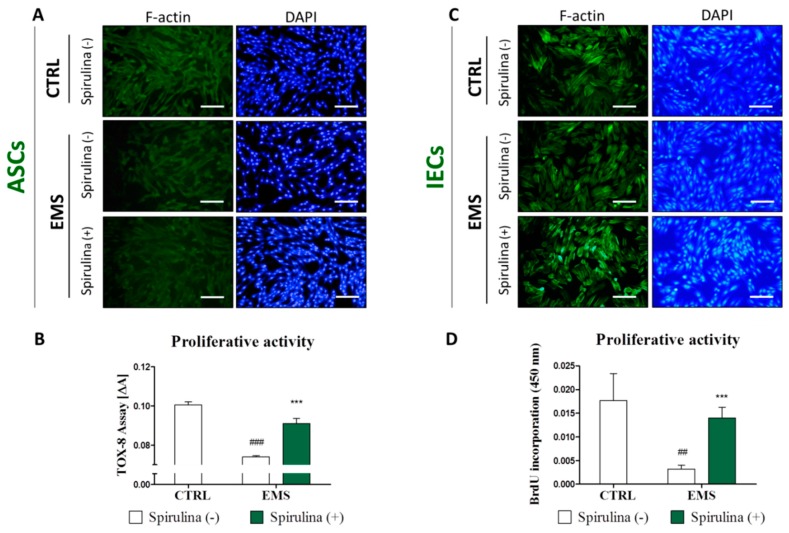
The effect of *Spirulina* treatment on equine adipose-derived mesenchymal stromal cells’ (ASCs’) and intestinal epithelial cells’ (IECs’) morphology and proliferative potential. Using fluorescence cell imaging technique allowed us to monitor the changes in the morphology of ASCs (**A**) and IECs (**C**) following exposition to *Spirulina platensis* aqueous extract. Actin filaments were visualized with atto-488-labeled phalloidin, the nuclei were counterstained with DAPI. Photographs presented in the graph are representative and show characteristic features of examined cultures. Cellular morphology of ASC_EMS_ and IEC_EMS_ was slightly altered in reference to the corresponding control cells, however, this effect was attenuated by treating cells with *Spirulina.* Proliferative potential of ASCs (**B**) was evaluated after 24 h of treatment by measuring the metabolic activity of cells manifested by bioreduction of resazurin dye. IECs proliferation (**D**) was measured by colorimetric quantification of DNA synthesized in the presence of BrdU label. A significant upregulation of proliferation was observed in both investigated cell populations cultured in the presence of *Spirulina platensis*. Magnification 100×, scale bars: 250 μm. Results expressed as mean ± SD, ^##^ *p* < 0.01, ^###/^*** *p* < 0.001.

Staining with DAPI and phalloidin revealed that ASC_CTRL_ exhibited rather bipolar fibroblast-like phenotype, whereas the majority of ASC_EMS_ cells were flat spread-out cells of irregular shape with enlarged nuclei. Additionally, ASC_EMS_ cultures attained lower confluence as compared to control culture. The exposure to Spirulina considerably improved morphology and growth pattern of ASC_EMS_. In Spirulina-treated cultures, increase in the number of elongated spindle-shaped cells with simultaneous reduction of flat multipolar cells was evident. Moreover, cells cultured in the presence of Spirulina grew more densely and reached significantly higher confluence after 48 h in culture, implying enhanced proliferative activity. Representative photographs are shown in Figure 2A.

IEC cells isolated from the small intestine of healthy horses exhibited a typical epithelial morphology of packed polygonal cells (Figure 2C), while IEC_EMS_ cells displayed slightly different phenotype manifested by increased epithelial separation reflected by shrunken cell bodies and wider, more frequent spaces between the cells. In turn, intercellular spaces of Spirulina-treated IEC_EMS_ appeared similar to those present in the IEC of the control group. Cellular adherence was more evident in this group indicating strengthen intercellular connections resulting from the exposure to Spirulina.

The growth rate of ASCs and IECs in vitro, after 24 h of Spirulina treatment was evaluated with resazurin-based TOX-8 assay (Figure 2B) or BrdU colorimetric assay (Figure 2D), respectively. Determination of cell viability in control cultures of ASCs and IECs derived from healthy or EMS horses revealed diminished proliferation in ASC_EMS_ and IEC_EMS_ cells when compared to counterparts isolated from healthy individuals, however 24-h exposure to Spirulina accelerated growth of cells. Cellular viability of both ASC_EMS_ and IEC_EMS_ was significantly restored following Spirulina supplementation (*p* < 0.001).

### 3.4. Spirulina Supresses Cellular Senescence and Apoptosis of Equine ASCs and IECs Derived from EMS-Diagnosed Horses

**Figure 3:**
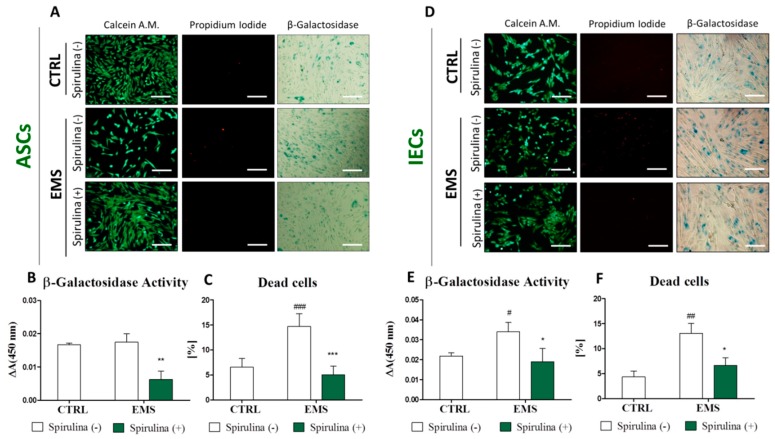
Cellular senescence and apoptosis level in equine adipose-derived mesenchymal stromal cells (ASCs) and intestinal epithelial cells (IECs) cultured under standard conditions or in the presence of *Spirulina* aqueous extract. Viable and dead cells were visualized in ASCs (**A**) and IECs (**D**) by simultaneous fluorescent staining with Calcein A.M. and Propidium Iodide, respectively. Percentage of dead cells within investigated cell populations was calculated on the basis of the image analysis performed on 10 representative pictures using ImageJ computer software (**C**,**F**). Moreover, senescent cells in cultures were identified (**A**,**D**) and spectrophotometrically quantified (**B**,**E**) on the basis of the expression of senescence-associated β-galactosidase. The results show that *Spirulina platensis* effectively inhibits apoptosis and senescence in ASCs and IECs derived from EMS-diagnosed horses. Magnification 100×, scale bars: 250 μm. Results expressed as mean ± SD, ^#/^* *p* < 0.05, ^##/^** *p* < 0.01, ^###/^*** *p* < 0.001.

To provide an explanation for enhanced growth of ASC_EMS_ and IEC_EMS_ induced by Spirulina extract supplementation, in the next step of the study we verified the relationship between proliferation and cell death/senescence. For simultaneous visualization of viable and dead cells in culture, Calcein A.M./Propidium Iodide double fluorescent staining was performed (Figure 3A,D). The ratios of dead cells within investigated populations were calculated on the basis of the image data analysis using ImageJ software. Results are presented in Figure 3C,F. In reference to the control groups of ASCs and IECs originating from healthy animals, cultures of ASC_EMS_ and IEC_EMS_ were characterized by substantially greater accumulation of necrotic (PI-positive) cells. A sufficient decline in the percentage of dead cells in ASC (*p* < 0.001) and IEC (*p* < 0.05) cultures was observed following Spirulina treatment.

A similar tendency was observed regarding the accumulation of β-galactosidase in cells. Qualitative and quantitative estimates of cellular senescence were provided using intended staining kit. Representative images showing blue-stained cells expressing β-galactosidase are presented in Figure 3A,D, in turn Figure 3B,E, show quantitative data obtained by spectrophotometric measurement of the dye absorption. There was an evident decrease in the abundance of cells expressing senescence-associated β-galactosidase within both ASC_EMS_ (*p* < 0.01) and IEC_EMS_ (*p* < 0.05) experimental groups cultured in the presence of Spirulina extract.

Taking all these data together, it is plausible to propose that *Spirulina platensis* stimulates proliferation and ameliorates the morphology of ASC_EMS_ and IEC_EMS_ by contribution to the rejuvenation of cells with accelerating-aging phenotype.

### 3.5. Quantitative Analysis of Apoptosis-Related Gene and p53 Protein Expression

**Figure 4:**
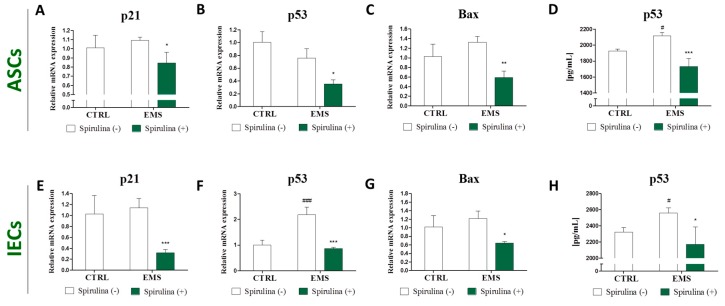
Apoptosis-related gene and p53 protein expression. Real time RT-PCR analysis of the expression of p21, p53 and Bax mRNA in equine adipose-derived mesenchymal stromal cells (ASCs) (**A**–**C**) and intestinal epithelial cells (IECs) (**E**–**G**) derived from control or EMS-diagnosed horses, cultured in the presence or absence of *Spirulina platensis*. The expression of apoptosis-related genes was significantly downregulated following *Spirulina* treatment. Additionally, p53 protein levels in supernatants from over cell cultures were investigated by ELISA (**D**,**H**). Results are expressed as mean ± SD, ^#/^* *p* < 0.05, ** *p* < 0.01, ^###/^*** *p* < 0.001.

In the next step of the study we assessed the expression of well-known apoptosis-inducing genes using real time RT-PCR. Results are shown in Figure 4. Quantitative analysis of the p21, p53, and Bax mRNA levels revealed no significant differences in the amount of the transcripts between control and EMS groups—both in the case of ASCs and IECs, except augmented p53 expression within IEC_EMS_ (*p* < 0.001) (Figure 4F). However, significant downregulation of p21 (Figure 4A,E), p53 (Figure 4B,F), and Bax (Figure 4C,G) was observed following Spirulina treatment in investigated cell populations, indicating lower susceptibility of those cells to apoptosis. Moreover, extracellular p53 protein secretion was analyzed quantitatively by ELISA. Results demonstrate that Spirulina treatment effectively suppressed upregulated p53 secretion in ASC_EMS_ and IEC_EMS_.

### 3.6. Spirulina Treatment Reduces Cellular Oxidative Stress and Protects Against Mitochondrial Dysfunction and Degeneration

**Figure 5:**
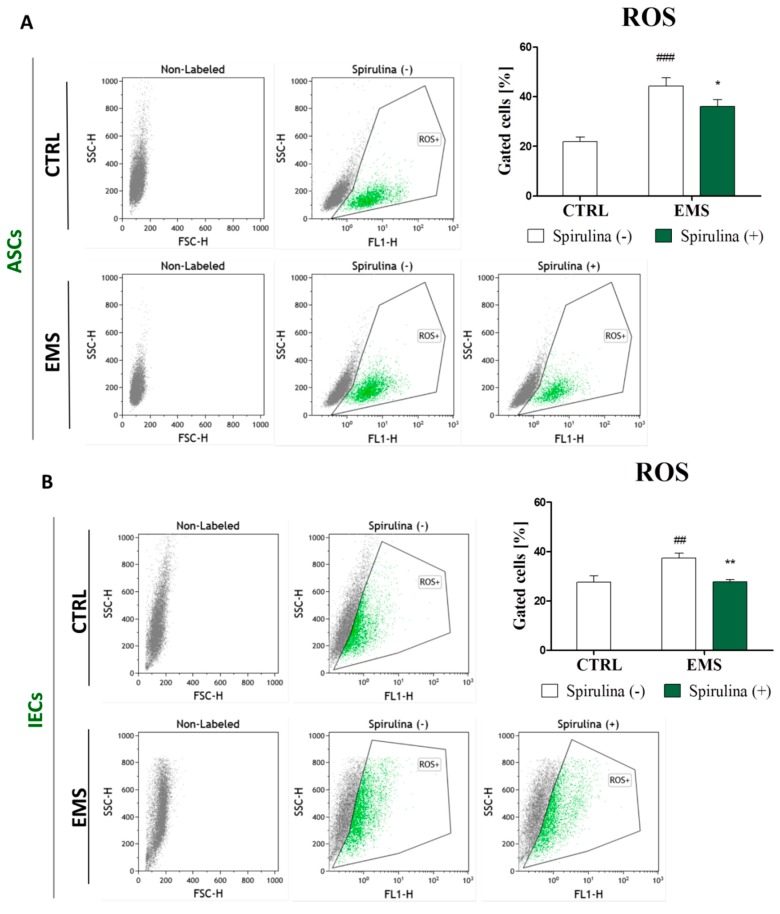
Accumulation of intracellular reactive oxygen species (ROS). To evaluate whether *Spirulina platensis* influences cellular oxidative stress level, intracellular ROS was detected in equine adipose-derived mesenchymal stromal cells (ASCs) (**A**) and intestinal epithelial cells (IECs) (**B**) with flow cytometry. The results show substantial reduction of elevated ROS in both ASCs and IECs isolated from EMS horses following exposition to *Spirulina platensis.* Dot plot graphs are provided as a visual representation of fluorescence-activated cell sorting (FACS) analysis. Quantitative data are displayed as box plots and expressed as mean ± SD, * *p* < 0.05, ^##/^** *p* < 0.01, ^###^ *p* < 0.001.

Senescence is characterized by an irreversible cell cycle arrest in response to various forms of stress, including DNA damage, oncogenic transformation, replicative stress, induction of ROS, and autophagy [28,29]. Hence, it was interesting to make an attempt to investigate whether Spirulina-stimulated reversibility of cellular aging and the following rejuvenation of ASCs and IECs may be due to overcoming oxidative stress and mitochondrial dysfunction. Detection of intracellular ROS by the means of flow cytometry revealed abundantly increased ROS levels in ASC_EMS_ (Figure 5A) and IEC_EMS_ (Figure 5B) when compared to counterpart populations originating from healthy individuals. Curiously, upon treatment with Spirulina, efficacious inhibition of upregulated reactive oxygen species (ROS) in both ASC_EMS_ (*p* < 0.05) and IEC_EMS_ (*p* < 0.01) experimental groups was observed.

**Figure 6:**
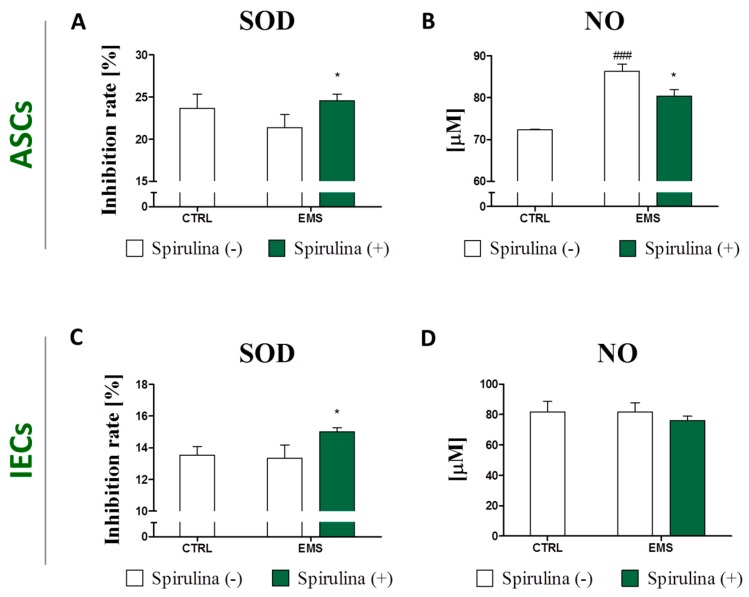
Changes in extracellular oxidative stress following treatment with *Spirulina platensis.* Quantification of secretory superoxide dismutase (SOD) was performed in cell-free culture supernatant of equine adipose-derived mesenchymal stromal cells (ASCs) (**A**) and intestinal epithelial cells (IECs) (**C**) in order to assess cellular antioxidant activity. Moreover, extracellular nitric oxide (NO) availability as a marker of oxidative stress was evaluated in culture media from over ASCs (**B**) and IECs (**D**). The data indicate reduction of extracellular stress level in response to *Spirulina platensis.* Results are expressed as mean ± SD, * *p* < 0.05, ^###^ *p* < 0.001.

In compliance with these data are results of the measurements of secretory superoxide dismutase (SOD) production, showing elevated cellular antioxidant activity of cells exposed to Spirulina (*p* < 0.05) (Figure 6A,C). Moreover, in the case of the ASC_EMS_ group, supplementation of culture media with Spirulina extract resulted in relevant downregulation of extracellular nitric oxide (NO) secretion (Figure 6B). Minor inhibition of NO production was likewise observed in Spirulina-treated IEC_EMS_ culture, however the difference between the experimental and non-treated control group was not statistically significant (Figure 6D).

**Figure 7:**
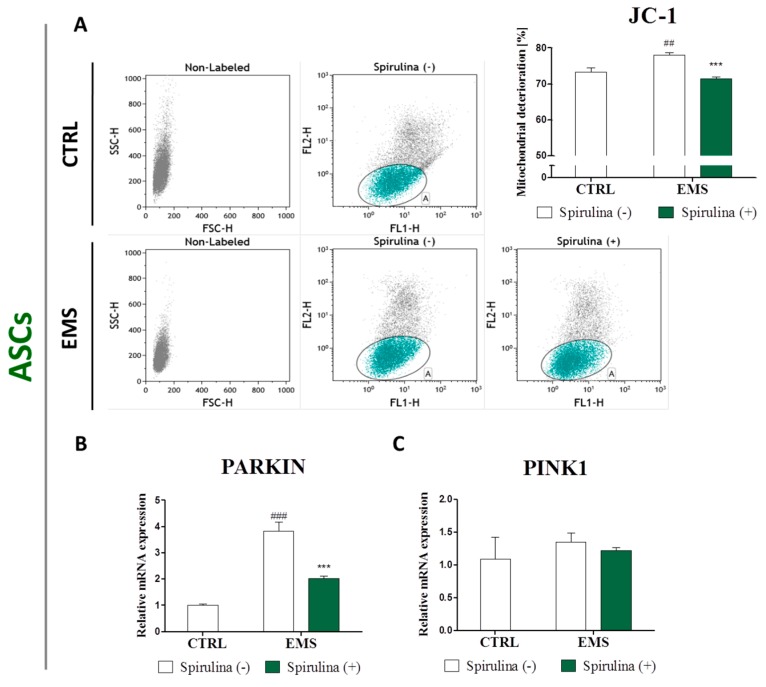

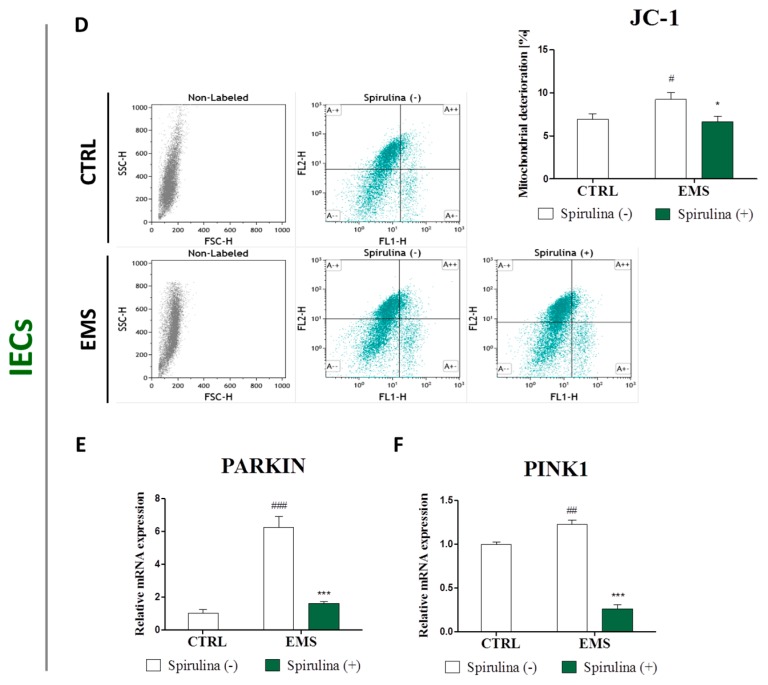
Evaluation of mitochondrial dysfunction and degeneration. JC-1 staining of equine adipose-derived mesenchymal stromal cells (ASCs) (**A**) and intestinal epithelial cells (IECs) (**D**) was performed to determine the effect of S*pirulina* treatment on mitochondrial quality and function. The percentage of deteriorated mitochondria was determined on the basis of green fluorescence emission. Moreover, expression of PINK1 (**C**,**F**) and Parkin (**B**,**E**) genes involved in mitochondrial quality control was quantified in ASCs (**B**,**C**) and IECs (**E**,**F**) using real time RT-PCR. Reduction of green fluorescence within the JC-1 stained cells alongside with downregulated PINK1 and Parkin expression may indicate for *Spirulina*-induced restoration of mitochondrial homeostasis. Dot plots constitute a visual representation of fluorescence-activated cell sorting (FACS) analysis. Quantitative data are expressed as mean ± SD, ^#/^* *p* < 0.05, ^##^ *p* < 0.01, ^###/^*** *p* < 0.001.

Additionally, to determine the effect of Spirulina treatment on mitochondrial function, JC-1 staining for cytometric assessment of mitochondrial membrane potential was performed. Moreover, expression of PINK1 and Parkin genes involved in mitochondrial quality control was quantified. The data are presented in Figure 7. Since mitochondrial disruption includes changes in the membrane potential due to the opening of the mitochondrial permeability transition pore, the abundance of impaired mitochondria could be assessed on the basis of the potential-dependent JC-1 dye accumulation in mitochondria [30,31]. The percentage of deteriorated mitochondria within investigated groups was examined by analyzing green fluorescence emission. A markedly increased mitochondrial dysfunction was observed in groups of ASC and IEC cells derived from EMS-diagnosed horses, when compared to those of healthy individuals. However, enrichment of culture media with Spirulina extract made a significant contribution to improvement of mitochondrial viability in ASC_EMS_ (*p* < 0.001) (Figure 7A) and IEC_EMS_ (*p* < 0.05) (Figure 7D). Furthermore, upregulated expression of Parkin in ASC_EMS_, as well as PINK1 and Parkin mRNAs in IEC_EMS_ was evident (Figure 7B,C,E,F). Cell culture in the presence of Spirulina resulted in significant inhibition of Parkin expression in ASC_EMS_ (*p* < 0.001) (Figure 7B) and IEC_EMS_ (*p* < 0.001) (Figure 7E), and PINK1 downregulation in IEC_EMS_ (*p* < 0.001) (Figure 7F).

### 3.7. Regulation of Nitric Oxide and Inflammatory Cytokines Production in LPS-Stimulated Murine Peritoneal Cavity Macrophages by Spirulina Platensis 

**Figure 8:**
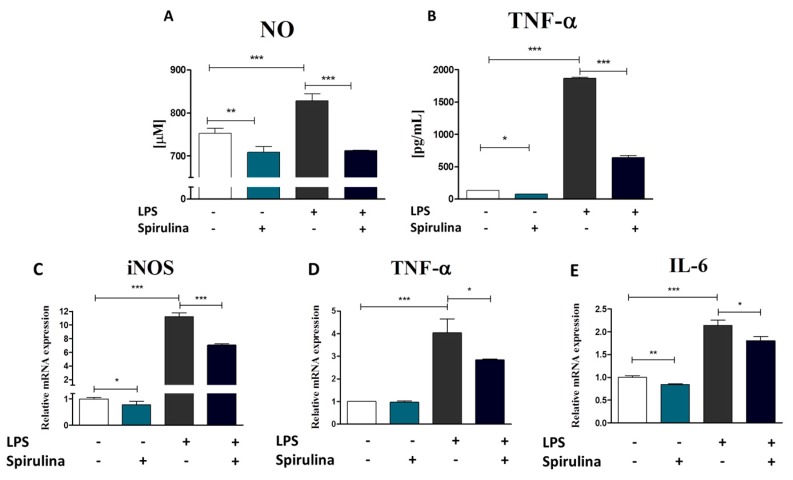
The effect of *Spirulina platensis* on the activation status of murine peritoneal macrophages. Mouse macrophages were isolated from the total pool of peritoneal exudate cells and cultured in the presence or absence of *Spirulina* water extract for 24 h. Macrophage inflammatory responses were subsequently triggered by LPS (1 µg/mL). Supernatants were collected and assayed for NO (**A**) and TNF-α (**B**). Moreover, the effect of *Spirulina* pre-treatment on iNOS (c), TNF-α (**D**) and IL-6 (**E**) gene expression was evaluated by the means of real time RT-PCR. The data show that *Spirulina* treatment effectively inhibited LPS-induced production of secretory NO and TNF-α, as well as overexpression of iNOS, TNF-α, and IL-6 in peritoneal macrophages. Results are expressed as mean ± SD, * *p* < 0.05, ** *p* < 0.01, *** *p* < 0.001.

For evaluation of the anti-inflammatory effect of S*pirulina platensis*, murine peritoneal cavity exudates were harvested as an easily accessible source of a variety of different immune cell populations [32]. Resident peritoneal macrophages were further purified from the total pool of cells exploiting their ability to adhere to culture surface. Macrophages represent a diverse population of mononuclear phagocytes that play a critical role in inflammation and its resolution [33]. Their activation accompanying inflammatory responses involves the production of two important inflammatory mediators: nitric oxide (NO) and tumor necrosis factor alpha (TNF-α) [34]. Therefore, changes in NO and TNF-α production by lipopolysaccharide (LPS)-activated or native macrophages in response to Spirulina treatment were investigated (Figure 8A,B). Our data show that cell pre-stimulation with Spirulina extract 24 h prior LPS treatment effectively inhibited LPS-induced macrophage activation and therefore NO (*p* < 0.001) (Figure 8A) and TNF-α (*p* < 0.001) (Figure 8B) production. Moreover, supplementation of culture media with Spirulina extract resulted in reduction of the basal level of NO (*p* < 0.01) and TNF-α (*p* < 0.05) in native cells as well (Figure 8A,B). To further verify whether Spirulina actually works at the molecular level through regulation of inflammatory and macrophage activation-associated gene expression, the levels of iNOS, TNF-α and IL-6 mRNAs were quantified. Results are presented in Figure 8C–E. A significant downregulation of the inducible nitric oxide synthase (iNOS) gene (*p* < 0.001) (Figure 8C) as well as pro-inflammatory TNFα (*p* < 0.05) (Figure 8D) and IL-6 (*p* < 0.05) (Figure 8E) cytokine expression was observed following exposure to Spirulina extract preceding LPS treatment. Considerable inhibition of iNOS and IL-6 genes expression was also observed in native cells cultured in the presence of Spirulina. The differences regarding untreated control cells were statistically significant in case of both genes (*p* < 0.05 and *p* < 0.01, respectively) (Figure 8C,E). Therefore, all of these data together clearly indicate anti-inflammatory effect of the *Spirulina platensis*.

### 3.8. Spirulina Platensis Reduces Insulin Resistance and Promotes Weight Loss in Equine Metabolic Syndrome-Affected Horses 

Body weight measurements revealed that all animals with diagnosed metabolic syndrome were obese and their body condition score (BCS) was rated 8–9 indicating extreme fat accumulation (Table 5). Three months of Spirulina supplementation resulted in significant weight loos. The mean body weight of EMS-horses fed with Spirulina dropped from 726.8 kg to 669.7 kg (*p* < 0.01). The reduction of BCS was also evident—from the average of 8.3 to 7.5 (*p* < 0.05). Another parameter related to obesity and taken into account when diagnosing EMS is cresty neck score (CNS), a system for assessing fat accumulation in the crest. The mean CNS was equal 2.0 for healthy horses involved into the experiment, and 4.0 for EMS-diagnosed horses. Spirulina supplementation resulted in reduction of this value up to 3.2 in horses with implemented dietary protocol (*p* < 0.001). Additionally, clinical examination revealed that Spirulina treatment resulted in reduced fasting insulin level (*p* < 0.05) and serum leptin concentrations, however, the difference between leptin level in serum of EMS horses before (8.6 ± 1.4 ng/mL) and after (8.3 ± 1.6 ng/mL) treatment was not statistically significant and still considerably higher than in healthy animals (2.9 ± 0.6 ng/mL). Moreover, prior to the experiment, combined glucose insulin test (CGIT) was performed for the evaluation of insulin dysregulation. All horses assigned to the healthy control group demonstrated negative CGIT results, while horses qualified for EMS-control or experimental groups positive. The test was repeated after the completion of dietary protocol. The results became negative for 5 out of 6 EMS-diagnosed horsed fed with pelleted Spirulina for three months. All together, these results clearly indicate improved insulin resistance in EMS-diagnosed horses following diet enriched with *Spirulina platensis*.

## 4. Discussion

Obesity, insulin resistance, and past/chronic laminitis are the most characteristic clinical signs of equine metabolic syndrome (EMS). In the course of the metabolic disorders, adipose tissue abundantly produces a wide range of hormones and pro-inflammatory cytokines as well as accumulates oxidative stress factors, which may play a fundamental role in the pathogenesis of EMS [35,36]. Elevated systemic and local inflammation, abundant accumulation of reactive oxygen species with simultaneously decreased superoxide dismutase activity, affects the both ASCs’ and IECs’ senescence, aging, and apoptosis [3,37]. Thus, searching for an effective stimulus that might enhance cellular viability, inhibit senescence, and improve proliferative potential of ASCs and IECs isolated from metabolic syndrome-affected individuals seem to be a promising therapeutic strategy. Spirulina has been widely reported to possess many beneficial therapeutic implications including arthritis, cardiovascular diseases, and diabetes [38]. Unique properties of this blue-green algae species result from the high content of nutrients such as proteins, phytochemicals, vitamins, minerals, carbohydrates, and γ-linolenic acid. Moreover, Spirulina has been shown to contain significant quantities of natural carotene and xanthophyll phytopigments, as well as phycocyanin, which due to their antioxidant, anti-inflammatory and anticancer properties have been identified as the most important active compounds of *Spirulina platensis* extracts, determining its therapeutic outcome [39]. Sanberg et al. [40] have reported that Spirulina effectively stimulates proliferation of stem cells, including bone marrow-derived progenitors, in a dose dependent manner. In turn, Deo et al. [41] have shown that Spirulina protects hippocampal neural progenitor cells’ proliferative potential from the insult of LPS-induced acute systemic inflammation. The protective effect of *Spirulina platensis* aqueous extract against apoptotic cell death induced by free radicals was also demonstrated in mouse 3T3 cells that were used as a model for epithelial cells [42]. The authors imply that reduced activation of apoptotic pathway results from the free-radical scavenging effect of phycocyanin, which is one of the most important functional constituents of *Spirulina platensis*. Phycocyanin derived from Spirulina has been further proved to diminish apoptosis in β-cells of pancreatic islets by enhancing antioxidant defense mechanisms [43]. Moreover, Ismail et al. [44] in their studies have shown that polysaccharides from *Spirulina platensis* improve nuclear enzyme function and DNA damage surveillance and repair mechanisms that are related to the chemoprevention of natural products. All mentioned above data stand in a good agreement with our current research findings, as we demonstrated that Spirulina water extract significantly improves proliferative activity and viability of both ASCs and IECs affected by metabolic syndrome by, inter alia, reducing their senescence manifested by β-galactosidase activity. Moreover, exposition of ASCs and IECs originating from EMS-diagnosed horses to Spirulina further resulted in inhibition of the expression of p21 and p53 which are widely considered as key apoptosis inducers involved in regulating cellular senescence progress and proliferation [45,46]. The observed rejuvenating and anti-apoptotic effect of Spirulina may be precisely due to the presence of C-phycocyanin, whose high content in water extract was reported [47]. Obtained data are consistent with those of Linjawi [48], who showed that Spirulina administration contributes to significant downregulation of p53, p21 and p27 in hepatic cells. Additionally, we observed reduced expression of Bax mRNA in ASC_EMS_ and IEC_EMS_ following Spirulina treatment. The protein encoded by this gene was reported to translocate to mitochondria upon induction of apoptosis, leading to the loss in mitochondrial membrane potential, and the release of cytochrome c. In consequence, cytochrome c triggers caspases activation, and programmed cell death occurs [49,50]. Therefore, reduction of Bax activation in cells impaired by metabolic syndrome may indicate the beneficial effect of Spirulina on mitochondrial function. Hence, in the next step of the experiment we evaluated mitochondrial membrane potential as an indicator of cells’ health and welfare within investigated populations. Flow cytometric analysis of JC-1 staining pattern provided information regarding mitochondria condition. Results show significantly increased number of de-energized mitochondria with decreased membrane potential in EMS-affected ASCs and IECs. However, this functional impairment of mitochondria was completely reversed in cells that had been cultured in the presence of Spirulina. Following this lead, we further investigated the expression of PINK1 and Parkin genes mediating mitochondrial biogenesis and dynamics. PINK1 and Parkin normally share signaling pathway and work together to identify impaired mitochondria and promote their elimination from mitochondrial network via autophagy. Upon mitochondrial damage, PINK1 accumulates on the outer membrane of mitochondria that lose membrane potential and activates Parkin E3 ubiquitin ligase that is subsequently recruited selectively from cytosol to dysfunctional mitochondria [51,52]. PINK1 and Parkin pathway has been previously suggested to be activated in response to metabolic stress in obese and diabetic subjects, to provide sufficient mitochondrial quality control [53,54]. Our data show considerably increased expression of both transcripts in IECs originating from EMS-diagnosed individuals. Moreover, robustly increased Parkin gene expression was evident in ASC_EMS_. Interestingly, supplementation of culture media with *Spirulina platensis* resulted in almost complete reversal of the impact of metabolic syndrome on PINK1 and Parkin genes. On the basis of these results, it is tempting to speculate that Spirulina treatment mediates PINK1/Parkin pathway downregulation and that this effect may be due to the protective function of Spirulina against metabolic stress induced mitochondrial injury and dysfunction. However, one limitation of our study is that PINK1 and Parkin expression was evaluated only at mRNA level and further investigation concerning mitochondrial co-localization of both proteins, as the marker for damaged mitochondria, is necessary to confirm our thesis. What is also important, we previously demonstrated that EMS condition affects ASCs’ function contributing to enormous accumulation of oxidative stress factors such as reactive oxygen species (ROS) and nitric oxide (NO), and so too downregulation of the activity of superoxide dismutase (SOD)—an enzyme that naturally protects the cells against the negative effect of free radicals. Moreover, serious mitochondrial disturbances including membrane raptures, disarrayed cristae, vacuole formation, as well as advantage of fission over fusion were observed in these cells [3,55,56]. In current research, we noticed reduced intracellular ROS and secretory NO accumulation alongside with increased SOD activity that suggest diminution of oxidative stress following exposition of ASCs and IECs derived from EMS horses to S*pirulina platensis*. There is an increasing number of studies indicating oxidative stress along with chronic low-grade inflammation as predisposing factors in the origin of metabolic syndrome [57,58]. In general, oxidative stress is a state of imbalance between oxidative and anti-oxidative cellular systems, and results in overproduction of reactive oxygen species (ROS) and reactive nitrogen species due to the insufficient mechanisms of antioxidant defenses such as superoxide dismutase (SOD). Under steady state conditions, SOD maintains oxygen related free radicals and reactive species, which are produced as by-products of aerobic metabolism, at low, non-toxic concentrations [59,60,61]. However, under various pathological conditions, when ROS overload exceeds the anti-oxidant capacity of cells, p53-mediated cell cycle arrest, DNA damage, and eventually cell death occurs [62]. In previous studies it was demonstrated that both ROS and NO, which are generated in excess in chronic inflammatory diseases of the gastrointestinal track, lead to the activation of critical cellular survival mechanisms in IECs [63,64]. It was also shown by Datta et al. [65] that persistent oxidative stress induced by heavy ion radiation correlates with mitochondria destabilization and activation of NADPH oxidase in mouse IECs, and leads to the induction of multiple DNA modifications and/or activation of apoptotic responses depending on the intensity of the radiation and time of the exposure. Moreover, elevated oxidative stress has been widely reported to affect mesenchymal stromal cells’ (MSCs’) longevity and functions addressing considerable repercussions on their therapeutic merit [66]. Therefore, reduction of oxidative stress in ASC_EMS_ and IEC_EMS_ in response to Spirulina treatment may likewise partially explain improved viability of those cells. Thus, it is worth emphasizing, that prevention of ROS and NO overproduction and induction of SOD scavenging activity constitutes another prerequisite for Spirulina treatment as an effective strategy for decreasing cell impairment. The antioxidant activity of *Spirulina platensis* water extract was demonstrated in the work of inter alia Santoyo et al. [67], who indicated zeaxanthin and myxoxanthophyll—the major carotenoids of Spirulina—together with the polar polyphenolic bioactive compounds, mainly responsible for its antioxidant capacity. Moreover, the antioxidant potential of *Spirulina platensis* against oxidative stress has been also attributed to the presence of C-phycocyanin—a selenium-containing biliprotein with a wide variety of pharmacological activities including superoxide and hydrogen peroxide radical-scavenging [42,68]. What is important in terms of our research is that, the free radical scavenging properties, and antioxidant activity of phycocyanin have been shown to contribute to its anti-inflammatory properties in both the in vitro model of inflammation [69] and in animal studies [70]. Since EMS is a metabolic disorder accompanied by widespread systemic inflammation [71], anti-inflammatory and immunomodulatory properties of Spirulina may be crucial considering its potential application. Our data show that Spirulina effectively suppressed LPS-induced inflammatory responses in macrophages isolated from murine peritoneal cavity. Inhibition of macrophage activation manifested by downregulation of LPS-induced NO and TNF-α protein as well as iNOS, TNF-α and IL-6 mRNA expression was evident following Spirulina treatment. These results are in a good agreement with previous studies of other research groups demonstrating anti-inflammatory action of *Spirulina platensis* [72,73,74,75,76,77].

In turn, when it comes to in vivo studies, our research indicates that a Spirulina-enriched diet not only accelerates weight loss in EMS-affected horses, but, what is most important, ameliorates insulin resistance. Clinical examination of individuals qualified to the experimental group subjected to 3-month dietary trial demonstrated reduced body mass, as well as improved serum insulin level and sensitivity. Our observations are consistent with those of inter alia Parikh et al. [78], who have proven the beneficial role of Spirulina supplementation in long-term glucose regulation and lipid homeostasis in type-2 diabetic patients.

Taking into account all the presented data, our research provides strong evidence for potential worthwhile health benefits resulting from *Spirulina platensis* application. Moreover, we believe that the engagement of *Spirulina platensis* nourishing as a complementary alternative approach may be helpful in supporting the conventional treatment of metabolic syndrome

## Figures and Tables

**Table 1:** Classification criteria for Equine Metabolic Syndrome.

Clinical Parameters								
No.	Group	Sex	Bw (kg)	BCS [1–9]	CNS [1–5]	Fasting insulin (mU/mL)	LEP (ng/mL)	CGIT:GLU in 45 min (mg/dL)
1	**Healthy**	F	610	6	1	7	3.21	74/n
2		F	644	7	2	12	4.12	69/n
3		F	627	7	2	9	2.87	71/n
4		M	609	6	1	8	1.86	89/n
5		M	649	7	2	14	3.56	80/n
6		M	639	6	2	13	2.91	74/n
	Mean ± SD		629.7 ± 15.7	6.5 ± 0.5	1.7 ± 0.5	10.5 ± 2.6	3.1 ± 0.7	76.2 ± 6.7
1	**EMS**	F	710	8	3	83	4.89	138/p
2		F	726	9	3	67	5.19	141/p
3		F	760	9	4	98	9.12	140/p
4		M	709	8	3	73	8.49	136/p
5		M	716	8	4	69	7.27	134/p
6		M	746	9	4	82	8.36	146/p
	Mean ± SD		727.8 ± 19.1	8.5 ± 0.5	3.5 ± 0.5	78.7 ± 10.5	7.2 ± 1.6	139.2 ± 3.8

F: female; M: male; Bw: body weight; BCS: body condition score; CNS: cresty neck score; CGIT: combined glucose-insulin test; SD: standard deviation; LEP: leptin; GLU: glucose; p: positive test results; n: negative test results.

**Table 2:** Sequences of primers used in real time polymerase chain reaction RT-PCR.

Gene	Sequence 5′—3′	Amplicon Length (bp)	Accession Number
p21	F: GAAGAGAAACCCCCAGCTCC R: TGACTGCATCAAACCCCACA	241	XM_003365840.2
p53	F: TACTCCCCTGCCCTCAACAA R: AGGAATCAGGGCCTTGAGGA	252	U37120.1
Bax	F: TTCCGACGGCAACTTCAACT R: GGTGACCCAAAGTCGGAGAG	218	XM_005596728.1
PINK1	F: GCACAATGAGCCAGGAGCTA R: GGGGTATTCACGCGAAGGTA	298	XM_014737247.1
Parkin	F: TCCCAGTGGAGGTCGATTCT R: CCCTCCAGGTGTGTTCGTTT	218	XM_005608126.2
GAPDH	F: GATGCCCCAATGTTTGTGA R: AAGCAGGGATGATGTTCTGG	250	NM_001163856.1
iNOS	F: GACAAGCTGCATGTGACATC R: GCTGGTAGGTTCCTGTTGTT	325	NM_001313922.1
TNF-α	F: ACAGAAAGCATGATCCGCGA R: CTTGGTGGTTTGCTACGACG	295	NM_013693.3
IL-6	F: GAGGATACCACTCCCAACAGACC R: AAGTGCATCATCGTTGTTCATACA	141	NM_001314054.1
β-Actin	F: CGACGATGCTCCCCGGGCTGTA R: CTCTTTGATGTCACGCACGATTTCCCTCT	574	NM_007393.5

F: sense primer; R: antisense primer; bp: base pair. p21: Cyclin Dependent Kinase Inhibitor 1A; p53: Tumor Suppressor p53; Bax: Bcl-2 associated X protein; PINK1: PTEN Induced Putative Kinase 1; Parkin: Parkin RBR E3 Ubiquitin Protein Ligase (PARK2); GAPDH: Glyceraldehyde-3-Phosphate Dehydrogenase; iNOS: inducible Nitric Oxide Synthase 2; IL-6: Interleukin 6; TNF-α: Tumor Necrosis Factor-α; β-Actin: PS1TP5-Binding Protein 1.

**Table 3:** Fatty acids content of *Spirulina platensis.*

Fatty Acid	Content [mg/100 g of Dry Mass]
8:0	5.1 ± 0.93
10:0	3.3 ± 0.57
12:0	12.9 ± 1.44
14:0	18.1 ± 1.01
14:1 (n-5)	n.d.
15:0	n.d.
16:0	2423.3 ± 241.11
16:1 (n-7)	282.7 ± 16.86
18:0	42.0 ± 6.13
18:1 (n-12)	173.0 ± 14.00
18:2 (n-6)	1053.3 ± 61.10
18:3 (n-3)	n.d.
18:3 (n-6)	744.0 ± 24.25
18:4 (n-3)	232.0 ± 27.87
20:0	12.6 ± 3.03
20:2 (n-6)	7.7 ± 0.87
22:0	n.d.

Results are expressed as the average of triplicate determinations ± standard deviation. 8:0: caprylic acid, 10:0: capric acid, 12:0: lauric acid, 14:0: myristic acid, 14:1 (n-5): myristoleic acid, 15:0: pentadecylic acid, 16:0: palmitic acid, 16:1 (n-7): palmitoleic acid, 18:0: stearic acid, 18:1 (n-12): coenzyme A, 18:2 (n-6): linoleic acid, 18:3 (n-3): alpha-linolenic acid, 18:3 (n-6): γ-linolenic acid, 18:4 (n-3): stearidonic acid, 20:0: arachidic acid, 20:2 (n-6): eicosadienoic acid, 22:0: behenic acid; n.d.: not detectable.

**Table 4:** Composition of free and protein-bound amino acids in *Spirulina platenis* (mg/100 g of dry mass).

Amino Acid	Free	Bound
Ala	207 ± 25.2	5167 ± 2757
Glc	97 ± 5.8	3657 ± 265.0
Val	50 ± 13.2	3320 ± 248.8
Leu	103 ± 5.8	4660 ± 390.0
Ile	42 ± 2.9	2760 ± 144.2
Asn	0	6367 ± 260.3
Asp	65 ± 5.0	
Gln	82 ± 7.6	7937 ± 361.2
Glu	437 ± 40.4	
Lys	35 ± 5.0	2530 ± 130.0
Arg	0	3477 ± 130.5
His	217 ± 20.8	763 ± 51.3
Phe	33 ± 2.9	2270 ± 175.2
Tyr	42 ± 7.6	2613 ± 263.5
Trp	0	757 ± 55.1
Ser	0	3297 ± 130.5
Thr	88 ± 7.6	1827 ± 115.9
Met	20 ± 0	1000 ± 87.2
Cys	0	540 ± 10.0
Pro	22 ± 2.9	1967 ± 151.8

During hydrolysis asparagine is converted to aspartic acid and glutamine to glutamic acid, therefore the sum of Asn + Asp and Gln + Glu is indicated as hydrolizate.

**Table 5:** Comparison of body weight, body condition score, cresty neck score, fasting insulin, leptin levels, and the results of combined glucose-insulin test before and after implementation of the dietary protocol including *Spirulina platensis* supplementation in EMS-diagnosed horses.

No.	Group	Sex	Clinical Parameters											
			Bw (kg)		BCS [1–9]		CNS [1–5]		Fasting Insulin (mU/mL)		LEP (ng/mL)		CGIT:GLU in 45 min (mg/dL)	
			Before Treatment	After Treatment	Before Treatment	After Treatment	Before Treatment	After Treatment	Before Treatment	After Treatmment	Before Treatment	After Treatment	Before Treatment	After Treatment
1	Healthy	F	580	592	6	6	2	3	2	6	3.21	3.13	78/n	75/n
2		F	550	565	6	6	2	2	4	3	2.89	3.78	69/n	73/n
3		F	590	575	6	6	2	2	2	2	3.14	2.98	72/n	75/n
4		M	559	562	6	6	2	3	7	5	2.91	2.47	81/n	77/n
5		M	528	543	6	6	2	2	5	4	3.38	2.94	79/n	71/n
6		M	560	551	6	6	2	2	3	3	2.71	2.01	67/n	73/n
	Mean ± SD		561.2 ± 22.0	564.7 ± 17.4	6.0	6.0	2.0	2.3 ± 0.5	3.8 ± 1.9	3.8 ± 1.5	3.0 ± 0.2	2.9 ± 0.6	74.3 ± 5.8	74.0 ± 2.1
1	EMS	F	760	748	9	9	4	4	78	92	10.41	9.48	132/p	118/p
2		F	732	728	9	8	4	4	91	83	9.29	9.22	126/p	129/p
3		F	743	751	9	9	4	4	87	80	9.89	10.31	149/p	138/p
4		M	708	712	9	9	4	4	74	73	8.78	9.13	121/p	119/p
5		M	719	720	9	9	4	4	86	82	7.98	8.43	138/p	131/p
6		M	698	692	9	9	4	4	92	89	8.13	7.88	136/p	129/p
	Mean ± SD		726.7 ± 23.0	725.2 ± 22.3	9.0	8.8 ± 0.4	4.0	4.0	84.7 ± 7.2	83.2 ± 6.7	9.1 ± 1.0	9.1 ± 0.8	133.7 ± 9.8	127.3 ± 7.6
1	EMS spirulina-treated	F	740	685	9	8	4	3	97	45	9.12	9.02	142/p	130/p
2		F	711	672	8	7	4	4	74	51	7.45	6.58	139/p	78/n
3		F	753	648	9	8	4	3	98	92	11.02	10.98	128/p	81/n
4		M	701	642	8	8	4	3	83	61	7.58	7.21	111/p	63/n
5		M	749	679	8	7	4	3	79	82	8.91	8.70	129/p	72/n
6		M	707	692	8	7	4	3	73	43	7.62	7.11	146/p	79/n
	Mean ± SD		726.8 ± 23.1	669.7 ± 20.3 **	8.3 ± 0.5	7.5 ± 0.5 *	4.0	3.2 ± 0.4 ***	84.0 ± 11.1	62.3 ± 20.4 *	8.6 ± 1.4	8.3 ± 1.6	132.5 ± 12.7	83.8 ± 23.5 **

F: female; M: male; Bw: body weight; BCS: body condition score; CNS: cresty neck score; CGIT: combined glucose-insulin test; SD: standard deviation; LEP: leptin; GLU: glucose; p: positive test results; n: negative test results. * *p* < 0.05, ** *p* < 0.01, *** *p* < 0.001.
